# A novel bedtime pulsatile-release caffeine formula ameliorates sleep inertia symptoms immediately upon awakening

**DOI:** 10.1038/s41598-021-98376-z

**Published:** 2021-10-05

**Authors:** Dario A. Dornbierer, Firat Yerlikaya, Rafael Wespi, Martina I. Boxler, Clarissa D. Voegel, Laura Schnider, Aslihan Arslan, Diego M. Baur, Markus R. Baumgartner, Tina Maria Binz, Thomas Kraemer, Hans-Peter Landolt

**Affiliations:** 1 Institute of Pharmacology and Toxicology, University of Zürich, Winterthurerstrasse 190, 8057 Zurich, Switzerland; 2 Sleep and Health Zurich, University Center of Competence, University of Zurich, Zurich, Switzerland; 3 Institute of Forensic Medicine, University of Zurich, Zurich, Switzerland; 4 Department of Pharmaceutical Technology, Lokman Hekim University, Ankara, Turkey; 5 Elixir Pharmaceutical Research and Development Corporation, Ankara, Turkey; 6 Department of Pharmaceutical Technology, Hacettepe University, Ankara, Turkey

**Keywords:** Neuroscience, Physiology

## Abstract

Sleep inertia is a disabling state of grogginess and impaired vigilance immediately upon awakening. The adenosine receptor antagonist, caffeine, is widely used to reduce sleep inertia symptoms, yet the initial, most severe impairments are hardly alleviated by post-awakening caffeine intake. To ameliorate this disabling state more potently, we developed an innovative, delayed, pulsatile-release caffeine formulation targeting an efficacious dose briefly before planned awakening. We comprehensively tested this formulation in two separate studies. First, we established the in vivo caffeine release profile in 10 young men. Subsequently, we investigated in placebo-controlled, double-blind, cross-over fashion the formulation’s ability to improve sleep inertia in 22 sleep-restricted volunteers. Following oral administration of 160 mg caffeine at 22:30, we kept volunteers awake until 03:00, to increase sleep inertia symptoms upon scheduled awakening at 07:00. Immediately upon awakening, we quantified subjective state, psychomotor vigilance, cognitive performance, and followed the evolution of the cortisol awakening response. We also recorded standard polysomnography during nocturnal sleep and a 1-h nap opportunity at 08:00. Compared to placebo, the engineered caffeine formula accelerated the reaction time on the psychomotor vigilance task, increased positive and reduced negative affect scores, improved sleep inertia ratings, prolonged the cortisol awakening response, and delayed nap sleep latency one hour after scheduled awakening. Based on these findings, we conclude that this novel, pulsatile-release caffeine formulation facilitates the sleep-to-wake transition in sleep-restricted healthy adults. We propose that individuals suffering from disabling sleep inertia may benefit from this innovative approach.

Trials registration: NCT04975360.

## Introduction

Sleep inertia is a disabling state of reduced physical and mental drive following awakening^1^, which typically lasts for less than 30 min but symptoms may persist for several hours in susceptible individuals^2–4^. A large portion of healthy adolescents report persistent difficulties to rise in the morning^5^ and many shift and on-call night workers exhibit impaired performance and grogginess after awakening, which raises important safety concerns in operational settings^6,7^. Furthermore, impaired post-awakening vigilance and mood is highly prevalent in a broad range of neurological and psychiatric conditions^7–10^.

Several biological and environmental factors influence the manifestation of sleep inertia. For example, abrupt awakening from deep sleep (also referred to as slow-wave sleep or stage N3 of non-rapid-eye-movement [NREM] sleep) is associated with more severe sleep inertia symptoms when compared to awakening from more superficial NREM sleep and rapid-eye-movement (REM) sleep^11,12^. This finding may suggest that neurophysiological processes underlying sleep and particularly deep NREM sleep carry over into wakefulness and contribute to behavioral and cognitive deficits associated with sleep inertia^13^. The proportion of deep sleep and the odds of awakening from N3 sleep is increased when a sleep opportunity is shorted such as in sleep restriction. Accordingly, abrupt awakening during sleep restriction enhances sleep inertia symptoms^4^.

The neuromodulator adenosine is a key regulator of deep sleep^14^ and adenosinergic neuromodulation may play an essential role in the manifestation of sleep inertia^15^. Consistent with this view, the adenosine receptor antagonist, caffeine, is widely used to counteract sleep inertia. Besides attenuating sleepiness and deficits in vigilance^16,17^, caffeine augments cardiovascular and respiratory functions^18^ and promotes the release of cortisol, a key hormone of the hypothalamic–pituitary–adrenal (HPA) axis^19^. The HPA axis regulates several psycho-vegetative aspects of the wake-up process, including the cortisol awakening response (CAR). The CAR reflects HPA axis function^20,21^ and may be associated with the propensity of sleep inertia^22^.

Because the impairments are most severe immediately upon awakening, proactive strategies aiming for optimal sleep length and timing have been recommended to minimize sleep inertia symptoms^6^. Nevertheless, it is not always possible to plan and obtain sleep of sufficient length and quality and at the optimal time of day. On the other hand, currently there exists no convincing evidence that reactive countermeasures to sleep intertia, i.e. strategies implemented upon wake-up, are sufficiently effective^6^. Although caffeine is the best available option, coffee takes 20–30 min to have an alerting effect^23^. The bioavailability of reactive oral caffeine intake prevents effective amelioration of sleep inertia symptoms for at least 12–18 min upon waking^17^, unless it is taken as a proactive countermeasure prior to a short sleep or nap bout^16,24^. When taken before sleep, however, caffeine can delay sleep onset, reduce total sleep time and attenuate the amount of deep slow wave sleep when a pharmacologically active concentration is present in the organism during sleep^24,25^.

The time-controlled administration of pharmaceuticals in accordance with the sleep–wake cycle provides an essential pillar in the emerging concept of chronopharmacology and chronotherapeutics^26,27^. We aimed at developing a chronotherapeutic caffeine formulation that ameliorates impaired subjective state, vigilance and performance immediately upon awakening without disturbing the quality of the preceeding sleep episode. For this purpose, we invented a delayed, pulsatile-release caffeine delivery system targeted to reach an efficacious plasma concentration approximately 7 h after intake. When ingested at habitual bedtime, we hypothesized that this formula would improve vigilance and mood, elevate the CAR, and reduce sleep propensity on the subsequent morning after wake-up from nocturnal sleep. We tested these hypotheses in two separate studies. First, we examined the in vivo release properties of the engineered caffeine formula throughout a nocturnal sleep episode. Then, we comprehensively investigated in randomized, double-blind, cross-over, placebo-controlled manner its effects on behavioral, emotional, neurocognitive and physiological symptoms of sleep inertia in sleep-restricted healthy young men.

## Methods

### Participants and permission

A total of 32 healthy young men (mean age: 25.6 ± 3.7 years) participated in the two studies (in vivo validation study: n = 10; pharmacodynamic study: n = 22), whereof 5 subjects participated in both experiments. The following criteria were required for inclusion: (i) male sex in order to avoid the potential impact of menstrual cycle on sleep physiology or HPA axis activity, (ii) age within the range of 18–34 years, (iii) a body-mass-index below 25, (iv) an Epworth Sleepiness Score (ESS) below 10, (v) habitual sleep onset latency below 20 min, (vi) regular sleep–wake rhythm with bedtime between 10 pm and 1 am, (vii) absence of any somatic or psychiatric disorders, (viii) no acute or chronic medication intake, (ix) non-smoking, (x) no history of drug abuse (lifetime use > 5 occasions, with exception of occasional cannabis use), and (xi) caffeine consumption of less than 4 units per day (coffee, tea, chocolate, cola, energy drinks).

The participants were instructed to abstain from illicit drugs and caffeine during the entire study, starting two weeks prior to the first experimental night until the end of the study (the day after the second experimental night). No alcohol was allowed 24 h before the expermental nights. The minimal wash out period before the experimental nights was 7 days. Participants were also instructed to keep an individual regular sleep–wake rhythm (23:00–07:00 or 22:00–06:00 depending on the volunteers’ habitual bedtime) during the entire study, starting 2 weeks prior to the experimental night. All included participants chose to keep either a 22:00–06:00 rhythm or a 23:00–07:00 rhythm. To ensure adherence to the regular sleep–wake pattern, participants were instructed to wear a rest-activity monitor on the non-dominant arm and to keep a sleep–wake diary.

The studies were approved by the Cantonal Ethics Committee of the Canton of Zurich (BASEC: 2018-00533) and registered on ClinicalTrials.gov (Identifier: NCT04975360). All participants provided written informed consent according to the declaration of Helsinki.

### Study drug

The caffeine pulsatile-release formulation was manufactured using a drug layering process^28^. The details of the engineering and manufacturing processes will be reported elsewhere. In brief, caffeine and the excipients were dispersed in the coating media and then sprayed onto inert microcrystalline cellulose spheres using a fluid bed through a Wurster tube with continuous inlet air that dries the liquid in the dispersion, to obtain various layers consisting of caffeine and release-controlling polymers. The applied release-controlling polymeric system was based on methacrylate copolymers, which control the release of caffeine in both a pH-dependent and pH-independent manner^29,30^. Thereby, the release mechanism of the polymeric system was mainly driven by the swellability and permeability of the copolymers^31^. The final micropellets were then encapsulated into hydroxy-propyl-methylcellulose capsules.

To evaluate the in vitro dissolution profiles of the manufactured formula, different prototypes were tested by means of state-of-the-art dissolution assays, mimicking gastrointestinal conditions^32,33^. Development and in vitro testing of the caffeine pulsatile-release formulation and placebos was conducted at Elixir Pharmaceutical Research and Development Corporation in Ankara. For the in vivo study, the most suited prototype with a favorable in vitro dissolution profile was chosen.

### In vivo validation study

In a first open-label evaluation study of the engineered delivery system, the in vivo caffeine release profile was determined in 10 fasted (no food or beverage consumption 2 h before drug administration) male individuals. After oral intake at 22:30, study participants were allowed to sleep from 23:00 to 07:00, while blood was continuously sampled. Samples were collected from the left antecubital vein at baseline (22:00), and 1.5, 2.5, 3.5, 4.5, 5.5, 6.5, 7.5, 8.5, 9.5, 10.5, 13.5 and 17.5 h after drug administration. During the sleep episode in the soundproof and climatized bedrooms of the sleep laboratory, the venous catheter was connected to a blood-collection setup in an adjacent room (Heidelberger plastic tube extensions through the wall). Thus, blood samples (4 ml, BD Vacutainer EDTA) were collected without disturbing the sleeping study participants. The intravenous line was kept patent with a slow drip (10 ml/h) of heparinized saline (1000 IU heparin in 0.9 g NaCl/dl; HEPARIN Bichsel; Bichsel AG, 3800 Unterseen, Switzerland). Blood samples were immediately centrifuged for 10 min at 2000 RCF and plasma samples were immediately stored on ice until final storage at − 80 °C.

### Pharmacodynamic study

In the pharmacodynamic study, the pulsatile-release caffeine formulation (which was validated in vivo as described above) or a placebo (matched in appearance) were administered 8.5 h before the scheduled wake-up time to the fasted participants (no food or beverage consumption 2 h before drug administration). To exacerbate sleep inertia symptoms and avoid neuropsychological ceiling/floor effects, which are frequent in intervention studies with highly functioning healthy volunteers, all participants were sleep restricted. More specifically, they were kept awake until 02:00 (participants adhering to a 22:00–06:00 rhythm) or 03:00 (23:00–07:00 rhythm), then given a 4-h sleep opportunity, and awoken at 06:00 (22:00–06:00 rhythm) or 07:00 (23:00–07:00 rhythm). At 3.5 h post-administration, all volunteers received a standardized, light meal. Blood was continuously sampled upon drug administration and the caffeine release from the formulation was monitored as described above. Upon awakening, the effects of the formulation on neurobehavioral, emotional, cognitive, and endocrinological markers of sleep inertia were assessed. Additionally, physiological sleep tendency was investigated by determining the sleep characteristics of a 1-h nap opportunity starting 1 h post-awakening. To simplify descriptions and data presentations, we will only refer to the 23:00–07:00 rhythm with respect to the time-points of the tasks, because only a small minority of the participants followed the 22:00–06:00 rhythm.

The pharmacodynamic study followed a randomized, double-blind, placebo-controlled, crossover design with a wash-out period of at least 1 week between the caffeine and placebo conditions.

**Figure 1:**
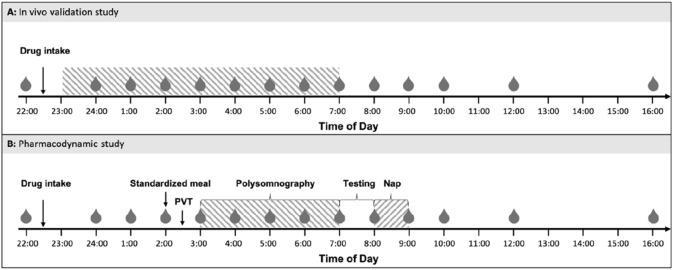
Illustration of the study procedures of the in vivo validation study (**A**) and the pharmacodynamic study (**B**). Timepoints of blood withdrawal are indicated as grey drops (drop symbols). Sleep episodes are highlighted as hatched areas. In both studies, sleep was continuously recorded by polysomnography. In the pharmacodynamic study, participants were kept awake until 3:00. Immediately after awakening from a restricted 4-h nocturnal sleep episode (at 07:00), volunteers performed a 1-h testing battery (referred to as “Testing”) to quantify behavioral, cognitive, emotional and physiological markers of sleep inertia. At 8:00, the participants were given a 1-h nap opportunity, while the latency to fall asleep and the sleep profile were recorded with polysomnography. Sleep inertia assessment, CAR measurements and physiological sleepiness testing were only performed in the pharmacodynamic study.

The details of both study designs are illustrated in Fig. 1.

### Analysis of caffeine levels

Caffeine and caffeine-^13^C_3_ were purchased from Sigma-Aldrich (St. Louis, USA). All chemicals used were of the highest purification grade available. Briefly, 200 µl of plasma, 50 µl of the internal standard (IS) (40 µM caffeine-^13^C_3_) and 50 µl of methanol (MeOH) were added to an Eppendorf tube. For protein precipitation, 400 µl of acetonitrile (ACN) were slowly added. Samples were shaken for 10 min and centrifuged at 10,000 rpm for 5 min. A volume of 350 µl of the supernatant was transferred into an auto-sampler vial and evaporated to dryness under a gentle stream of nitrogen. For reconstitution, 250 µl of an eluent-mixture (95:5, v/v) was added. Quality control (QC) samples and calibrators (Cal) were prepared with the same sample preparation, replacing the 50 µl of MeOH with the Cal or QC solutions. The plasma samples were analyzed on an ultra-high performance liquid chromatography (UHPLC) system (Thermo Fisher, San Jose, CA, USA) coupled to a linear ion trap quadrupole mass spectrometer 5500 (Sciex, Darmstadt, Germany). The mobile phases of the UHPLC consisted of water (eluent A) and a mixture (70:30 v/v) of MeOH and ACN (eluent B), both containing 0.1% of formic acid (v/v). A volume of 5 µl of the prepared samples was used for quantification. Using a Kinetex Biphenyl column (50 × 2.1 mm, 1.7 µm) (Phenomenex, Aschaffenburg, Germany), the flow rate was set to 0.45 ml/min with the following gradient: start conditions 95% of eluent A, decreasing to 80% in 3 min followed by a quick decrease to 2% within 0.5 min. These conditions were held for 1 min and switched to the starting conditions for re-equilibration for 1 min. The mass spectrometer was operated in positive electrospray ionization mode with scheduled multiple reaction monitoring. Three MRM transitions were used for both analytes. For quantification, the peak area of the analytes was further integrated and divided by the peak area of the IS. Cal samples were fitted with a least-squares fit and weighted by 1/x. The limit of quantification of caffeine was 1.2 µM.

### Testing of neurobehavioral performance and subjective state

A comprehensive test battery including the following tasks, validated questionnaires, and physiological measures to assess behavioral, subjective, emotional, cognitive and endocrinological markers of sleep inertia was administered immediately upon awakening (07:00–08:00).

### Psychomotor vigilance test (PVT)

Vigilance was assessed with a 10-min version of the PVT^34^ at 02:30 and 07:05 am, i.e. immediately after awakening. The median reaction time (RT) and the numbers of lapses (trials with RT > 500 ms) were analyzed.

### Positive and negative affective schedule (PANAS)

The PANAS^35^ was used to assess mood 15 min after awakening (at 07:15 am).

### Caffeine acute questionnaire (CAQ)

To assess specific caffeine-related effects, the CAQ^36^ was applied 30 min after awakening. Participants were asked to rate the following items on a 5-point scale (not at all, a little bit, moderate, much, very much); “Are you feeling any of these caffeine-related effects”: “increased vigilance”, “increased wellbeing”, “increased heart rate”, “increased sociability”, “increased motivation to work”, “increased tension”, “increased concentration”, “increased urge to urinate”, “increased shakiness”, “reduced appetite”, “increased energy”, “increased sweating”, “increased self-confidence”, “reduced headache”, “increased anxiety”, “reduced tiredness”, “stimulation”, “increased nervousness”, “reduced boredom” and “increased stomach trouble”.

### Modified sleep inertia questionnaire (SIQ)

We modified the SIQ^37^ to assess volunteers’ subjective experience of the awakening process. The original version of the SIQ represents a trait inventory, in which participants are instructed to rate the quality of their awakening process during the last week, namely on physiological, emotional, cognitive and behavioral levels. Thereby, the inventory instruction reads as follows: “On a typical morning in the past week, after you woke up, to what extent did you, for example, have problems to get out of bed” (possible ratings: 1 = not at all, 2 = a little, 3 = somewhat, 4 = often, 5 = all the time)*.* For the present study, we rephrased the inventory’s instruction to gain state information of the wake-up process of the experimental morning (rather than trait information of the last week), to analyze the acute effects of our pulsatile-release formula. The instruction was rephrased as follows*: *“How strong did you feel the following aspects after you woke up this morning compared to a normal morning last week: for example, have problems to get out of bed” (possible ratings: − 3 = extremely less, − 2 = much less, − 1 = a little bit less, 0 = same, 1 = a little bit more, 2 = much more, 3 = extremely more). For our purpose, the modified version of the SIQ was renamed to Acute Sleep Inertia Questionnaire (ASIQ) and was administered at 07:45.

### N-back task

At 7:47 the n-back task was executed in 1-, 2- and 3-back versions^38^. Over a period of 7 min, a random series of letters were displayed and subjects were instructed to press a key when the currently displayed letter corresponded to the previous (1-back), the penultimate (2-back) or the antepenultimate (3-back) letter, respectively. The reaction times and the number of correct and incorrect answers were assessed.

### d2-task

Finally, the d2-task, a neuropsychological measure of selective and sustained attention and visual scanning speed^39^, was administered at 07:55. In this task, participants were instructed to cross out any letter “d” with two marks above it or below it in any order. The surrounding distractors were either a “p” with two marks or a “d” with one or three marks. For each line (14 lines in total), subjects were given 20 s to mark all “d” with two marks and then instructed to proceed to the next line. The number of correct and incorrectly crossed characters were determined.

All questionnaires as well as the n-back and d2 tasks were administered as paper–pencil versions.

### Cortisol awakening response (CAR)

Cortisone-D_7_ was purchased from Sigma Aldrich (Buchs, Switzerland) and ^13^C_3_-cortisol was purchased from Isoscience (Ambler, USA). Saliva of each subject was sampled at time points 07:00 (immediately after awakening), 07:15, 07:30, 07:45, and 08:00. Participants were instructed to chew the swab for 60 s and then return it into the Salivette^®^ tube (Sarstedt, Germany). After sampling, tubes were immediately stored on ice until final storage at − 80 °C. For cortisol detection, tubes were defrosted and centrifuged for 5 min at 5000 rpm to yield clear saliva in the conical tube. Two subjects had to be excluded, as the amount of saliva yielded from the swabs was insufficient. Then, the swab was removed and the yielded saliva was spiked with 50 μl IS (0.1 ng/μl Cortison-d_7_) for further analysis. A fully automated supported liquid extraction (SLE) was carried out by transferring 265 μl saliva into a column rack (24 × 6 ml) from Biotage^®^ Extrahera (Biotage, Uppsala, Sweden) and adding 300 μl water to the sample. After mixing the extracts were automatically loaded onto Isolute SLE + columns and allowed to absorb for 5 min. Analytes were then eluted two times with 1.5 ml ethyl acetate with a waiting time of 5 min in-between. The extracts were dried in a Turbovap^®^ (Biotage, Uppsala, Sweden) at 35 °C. The dry residues were resuspended using 150 μl methanol and 350 μl ammonium formate (5 mM) solution, which was used for liquid chromatography-tandem mass spectrometry (LC–MS/MS) analysis following a recently published method using ^13^C_3_-labeled cortisol as surrogate analyte for calibration^40^. The saliva samples were analyzed on an LC–MS/MS system that consisted of a Shimadzu Prominence UFLC (Shimadzu, Kyoto, Japan) high pressure liquid-chromatography (HPLC) system coupled to a Sciex QTRAP^®^ 6500 + linear ion trap quadrupole mass spectrometer (Sciex, Darmstadt, Germany). 10 μl of the samples were injected onto a Phenomenex® Kinetex® C_18_ column (2.6 μm, 50 × 2.10 mm). The mobile phase consisted of 10 ml ammonium formate (1 M) and 2 ml formic acid in 2 l water (A) and 10 ml ammonium formate (1 M) in 2 l methanol (B). The flow rate was 0.3 ml/min and the temperature of the column oven was set to 40 °C. The quantification was achieved by using the mass spectrometer in multiple reaction monitoring (MRM) with an ion spray voltage of − 4500 V. Cortisol was measured as formic acid adduct [(M–H) + 46]^−^ in negative electrospray ionization mode. The method was validated according to the guidelines of the German Society of Toxicology and Forensic Chemistry (GTFCh). The calibration was prepared by adding ^13^C_3_-cortisol to saliva in the concentration range of 0.55 nmol/ml up to 55 nmol/ml. QC samples were prepared in low concentrations (1.5 nmol/l). The limit of detection for cortisol was 0.55 nmol/l and the limit of quantification was 1.1 nmol/l.

### Polysomnography

As in previous studies^41–44^, sleep in vivo from 03:00 to 07:00 in the pharmacodynamic study was quantified by all-night polysomnography with Rembrandt^®^ Datalab (Version 8; Embla Systems, Planegg, Germany). in vivo Sleep pressure after awakening was also assessed by determining the participants’ sleep onset latency (SOL) and sleep patterns during a nap opportunity starting at 08:00 (Fig. 1). The recording setup consisted of 10 EEG electrodes (Fp1, Fp2, F3, F4, C3, C4, P3, P4, O1, 02) according to the 10–20 system^45^, a bipolar electrooculogram (EOG), a submental electromyogram (EMG) and an electrocardiogram (ECG). The individual EEG electrode coordinates were marked by cutting a few hairs at the electrode positions, to ensure that the electrodes were placed at the very same place in both experimental conditions.

All data were recorded with dedicated polysomnographic amplifiers (Artisan^®^, Micromed, Mogliano Veneto, Italy). As in previous studies of the lab (Dornbierer et al. 2019), the analog signals were conditioned by a high-pass filter (EEG: − 3 dB at 0.15 Hz; EMG: 10 Hz; ECG: 1 Hz) and an antialiasing low-pass filter (− 3 dB at 67.2 Hz), digitized and stored with a resolution of 256 Hz (sampling frequency of 256 Hz). The data of one participant were excluded from the nocturnal sleep analyses, due to insufficient EEG quality.

### Visual sleep stage scoring

Sleep variables were visually scored based on 30-s epochs according to the criteria of the American Academy of Sleep Medicine^46^. For sleep scoring, the C3-A2 derivation was used. Movement- and arousal-related artifacts were visually identified and excluded from the analyses. The following sleep variables were computed: time spent in (i) wakefulness (Wake), (ii) stage 1 (N1), (iii) stage 2 (N2), (iv) stage 3 (N3), (v) stage REM sleep, (vi) sleep onset latency (SOL = time between lights-off and first occurrence of N1), (vii) sleep efficiency (SEFF = [TST⁄TIB]*100%; TST = time spent in N1, N2, N3 and REM sleep; TIB = time between lights-off and lights-on), and (viii) pre-awakening stages wake, N1, N2, N3 and REM sleep.

### Statistical analyses

Independent linear mixed-effects models, with condition (caffeine *vs.* placebo) and time point (02:30 and 07:05 for PVT analyses; 07:00, 07:15, 07:30, 07:45 and 08:00 for CAR analyses) as within-subject factors, and subject ID as random effect were employed on ‘R’ for the analysis of the (1) PVT; (2) PANAS; (3) CAQ; (4) ASIQ; (5) n-back; (6) d2 task; (7) CAR; (8) nocturnal sleep variables; and (9) morning nap variables (RStudio Version 1.0.136; RStudio, Inc.; R-package “lme4,” Version 1.1–15). For all applied models, normal Q-Q plots were applied, demonstrating normality of the residuals. Moreover, the assumption of homoscedasticity and linearity was verified using a Tukey-Anscombe plot (residuals vs. fitted). Post-hoc testing was carried out using the ‘R’ package *emmeans* (Version 1.2.1). The *p* values of the post-hoc tests were corrected for multiple comparison using Benjamini–Hochberg correction of the false discovery rate^47^. If not noted otherwise, only significant effects and differences are reported.

## Results

### Caffeine release profiles

**Figure 2:**
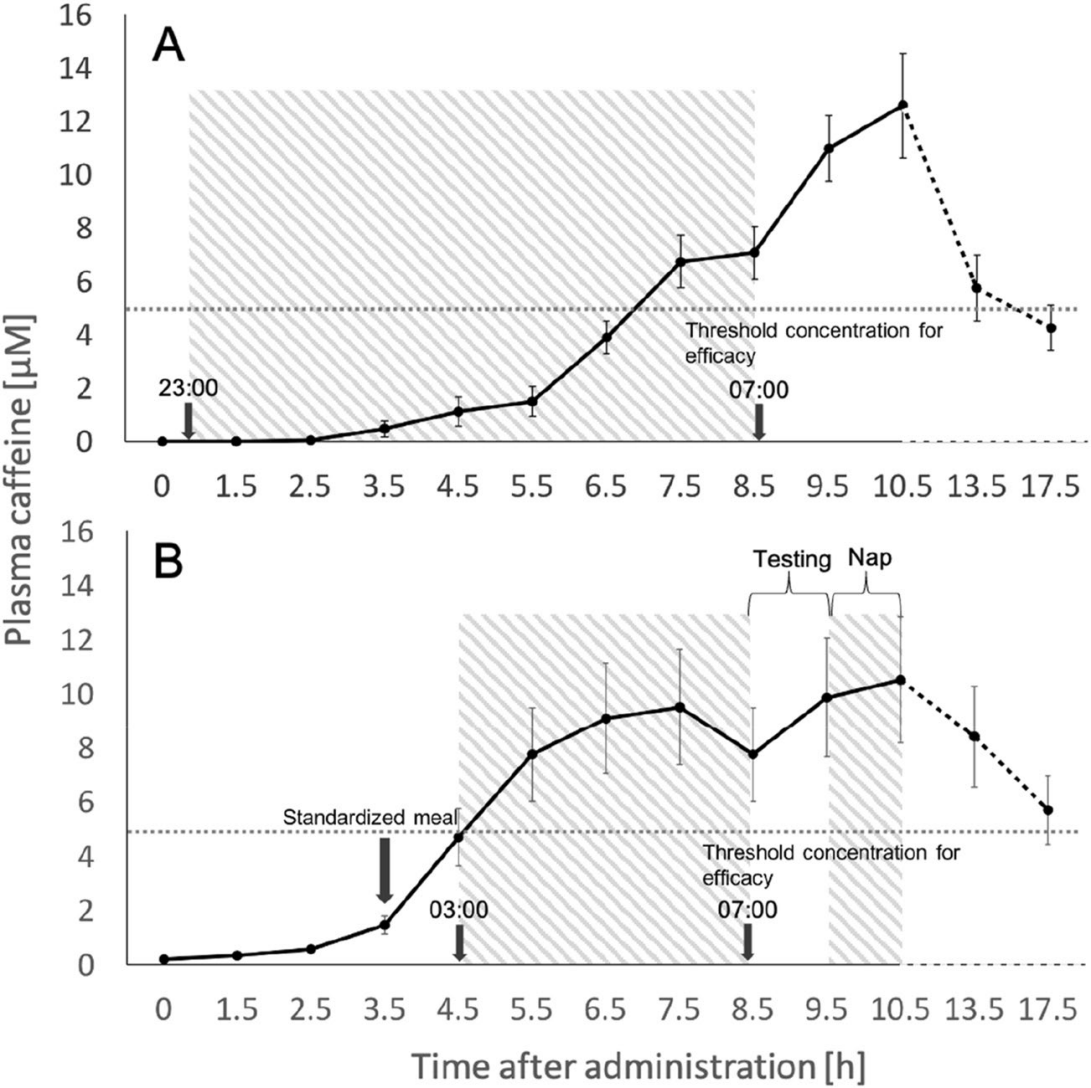
Evolution of the caffeine plasma concentration over time for the in vivo validation study (**A**) and the pharmacodynamic study (**B**). Black dots indicate mean caffeine plasma concentrations, error bars indicate standard errors (SEM). The horizontal dashed line at 5 μM indicates the threshold concentration of caffeine efficacy. Time point ‘0’ on the x-axis refers to 22:30 when the caffeine was administered. Sleep periods are indicated as hatched areas.

The in vivo validation study during sleep revealed a pulsatile-release profile of the administered formulation; c_max_ (maximal plasma concentration) was reached after 10.5 h (Fig. 2A). The caffeine curve followed a sustained-release profile, and efficacious plasma levels (> 5 μM) were attained after 7 h^48,49^.

Unexpectedly, in the pharmacodynamic study, the caffeine release profile distinctly differed from that in the in vivo validation study. A sustained-release of caffeine started after 3.5 h and efficacious plasma caffeine levels were already attained 5 h post-administration (Fig. 2B). The premature burst was most likely triggered by gastric movements due to a standardized meal that was served to the study participants 3.5 h post-administration in the pharmacodynamic study. This meal was absent in the in vivo validation study.

### Neurobehavioral, subjective, emotional and physiological symptoms of sleep inertia

#### Psychomotor vigilance test (PVT)

**Figure 3:**
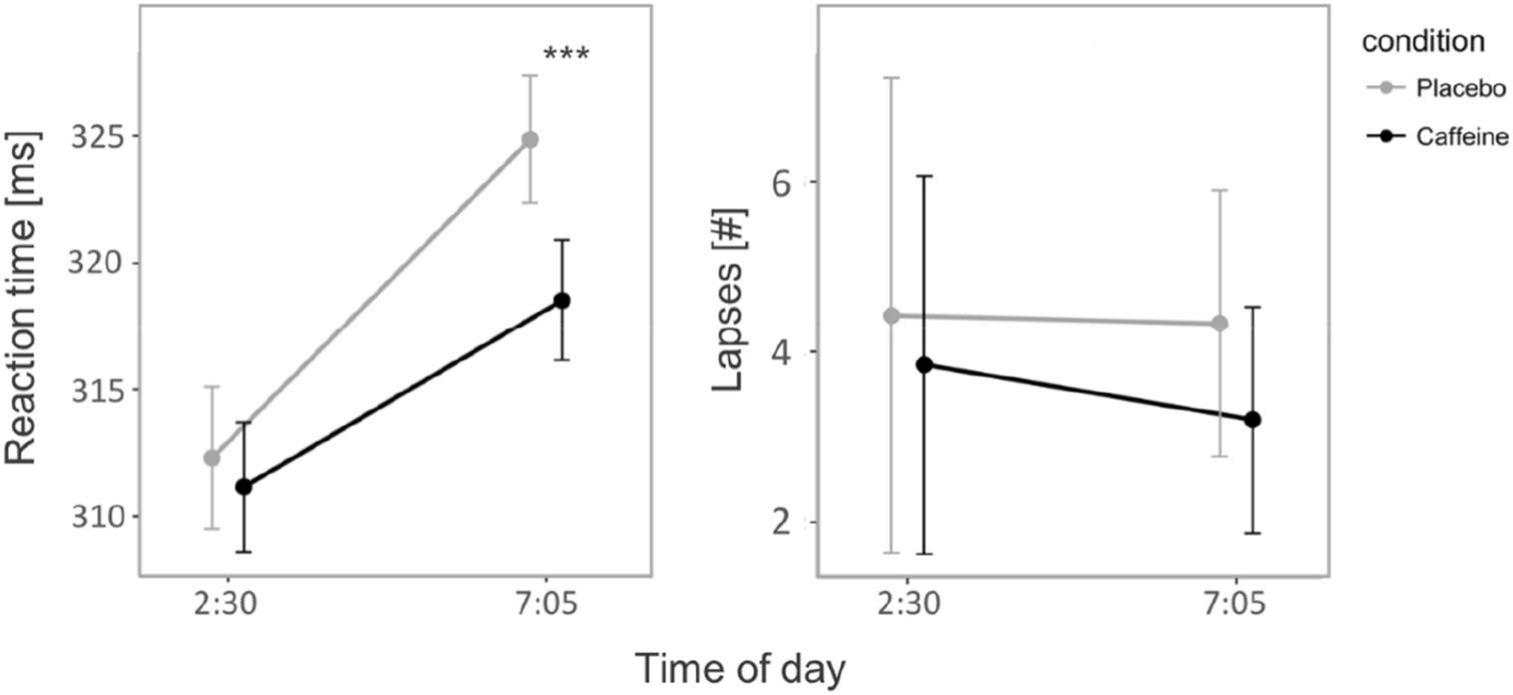
Median PVT reaction time (left) and number of lapses (right) at 2:30 and 7:05. Mean values (dots) and standard error of the mean (vertical lines) are shown. Grey lines indicate the placebo condition; black lines indicate the caffeine condition. ***p < 0.001 (Benjamini–Hochberg corrected).

The statistical analyses of the PVT data revealed a significant condition * timepoint interaction for median reaction time (F = 29.45; *p* < 0.001), such that caffeine improved the PVT median response time by roughly 10 ms when compared to placebo (Fig. 3). On the other hand, the number of lapses remained unaffected (F = 1.04; *p* = 0.359).

#### Positive–negative affect Scale (PANAS)

**Figure 4:**
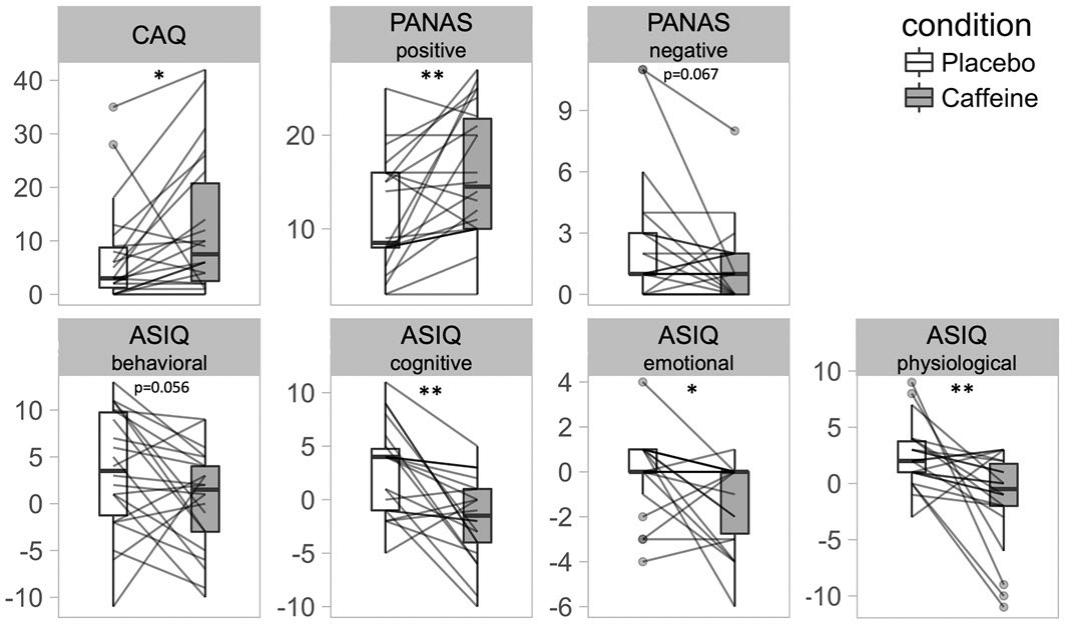
Post-awakening (7:15–8:00) assessments of subjective state. ASIQ = Acute Sleep Inertia Questionnaire (administered at 07:45). CAQ = Caffeine Acute Questionnaire. PANAS-positive = positive affective scale (administered at 07:15). PANAS-negative = negative affective scale (administered at 07:30). *p < 0.05; **p < 0.01 (Benjamini–Hochberg corrected).

Statistical analyses of the PANAS ratings revealed a significant condition * item interaction (F = 8.49; *p* = 0.004; η^2^ = 0.118), such that the engineered caffeine formulation increased positive ratings (*p* < 0.01) and tended to reduce negative ratings (*p* = 0.067) when compared to placebo (Fig. 4).

#### Caffeine acute questionnaire (CAQ)

The statistical analyses revealed significantly increased CAQ ratings in the caffeine condition when compared to placebo (F = 5.14; *p* = 0.034; η^2^ = 0.196; Fig. 4).

#### Acute sleep inertia questionnaire (ASIQ)

The statistical analyses revealed a significant condition effect (F = 31.21; *p* < 0.001; η^2^ = 0.175), such that the engineered caffeine-release formula reduced the ratings on all subscales of the ASIQ (behavioral, cognitive, emotional; physiological; Fig. 4). Remarkably, several individuals reported less problems to rise from bed on the experimental mornings compared to a normal morning in the week preceding the experiment (indicated as negative values in Fig. 4). This notion was particularly true during the caffeine condition (n = number of subjects with negative values: *behavioral* n = 9, *cognitive* n = 12, *emotional* n = 9, and *physiological* n = 10) and to a minor degree also during the placebo condition (*behavioral* n = 5, *cognitive* n = 7, *emotional* n = 5, and *physiological* n = 2).

#### N-back and d2 tasks

The statistical analyses of the n-back working memory (F = 0.43; *p* > 0.05) and the d2 sustained-attention tasks performance (F = 0.29; *p* > 0.05; η^2^ = 0.001) revealed no significant condition effects (data not shown).

### Cortisol awakening response (CAR)

**Figure 5:**
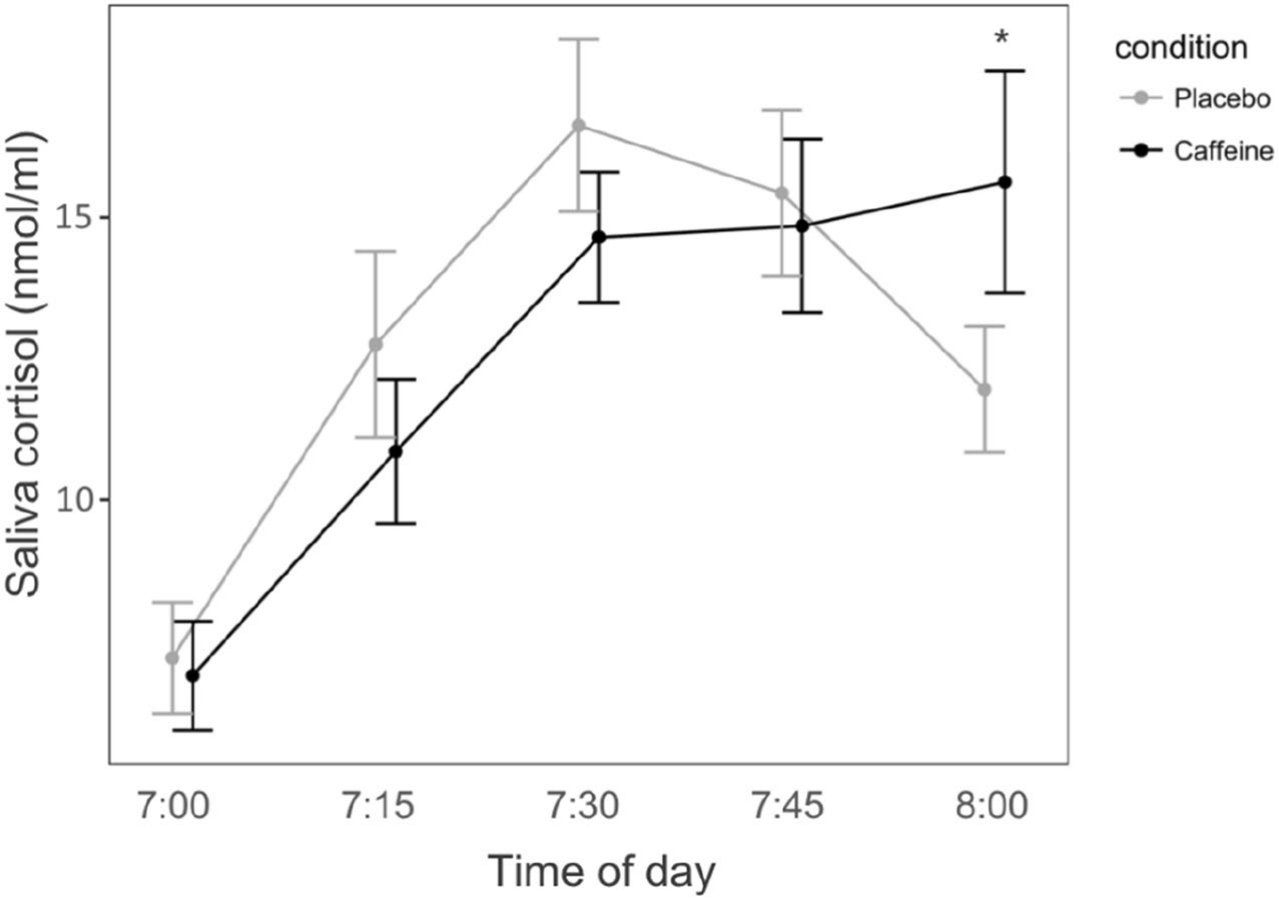
Salivary cortisol awakening response (CAR). Mean salivary cortisol concentration (dots) and standard error of the mean (vertical lines) are shown. Grey lines indicate the placebo condition; black lines indicate the caffeine condition. *p < 0.05 (Benjamini–Hochberg corrected).

The statistical analyses of the salivary cortisol levels revealed no significant main effect (*p* > 0.05). Nevertheless, post hoc testing revealed significantly increased cortisol levels at 08:00 (60 min post-awakening) in the caffeine condition when compared to placebo condition (t = 3.00; *p* < 0.04; Fig. 5).

### Sleep characteristics

#### Nocturnal sleep

**Figure 6:**
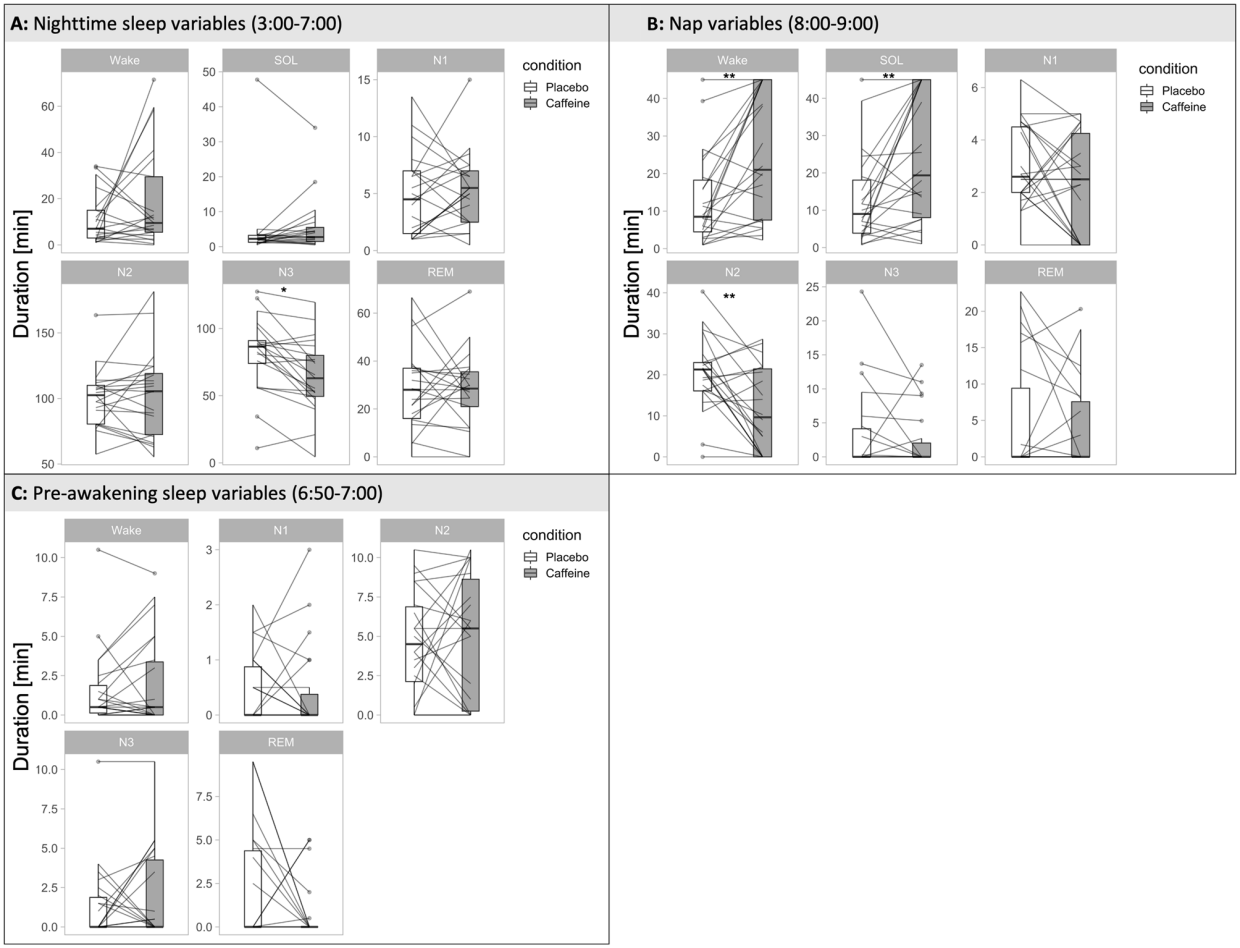
Visually-sored sleep variables in (**A**) the 4-h nighttime sleep episode (03:00–07:00); (**B**) the final 10 min before scheduled awakening (06:50–07:00); and (**C**) the 1-h nap sleep opportunity (08:00–09:00) are shown. SOL, sleep onset latency; N1-3, non-rapid-eye-movement sleep stages N1-3; REM, rapid-eye movement sleep; *p < 0.05; **p < 0.01 (Benjamini–Hochberg corrected).

Given that the duration of wakefulness was experimentally prolonged prior to the initiation of nocturnal sleep, in both conditions, participants showed a sleep onset latency shorter than 10 min and more than 2 h of deep stage N3 sleep. For all sleep variables analyzed (Table 1), the linear mixed-effects models revealed no significant main effect of “condition” (F = 0.56; *p* > 0.05; η^2^ = 0.0025). Nevertheless, post hoc testing with the ‘R’ package *emmeans* indicated that the time spent in N3 sleep was shorter (*p* = 0.004) in the caffeine condition when compared to the placebo condition (Fig. 6A; Table 1).

**Table 1:** Visually-scored sleep variables.

	Variable	Placebo		Caffeine		df	t-value	*p* value
		Mean	SD	Mean	SD			
Nocturnal sleep	Wake	11.17	10.78	19.03	21.70	20	1.51	0.439
	SOL	4.41	9.99	5.71	7.73	20	1.08	0.583
	N1	4.86	3.67	5.41	3.25	20	0.64	0.674
	N2	100.17	22.34	103.29	32.96	20	0.59	0.674
	N3	81.60	27.61	64.43	27.58	20	− 4.05	0.004
	REM	28.26	17.28	27.93	16.05	20	− 0.09	0.933
Pre awakening	Wake	1.57	2.48	1.96	2.89	21	0.88	0.650
	N1	0.43	0.62	0.41	0.81	21	− 0.13	0.898
	N2	4.57	3.36	5.00	3.99	21	0.48	0.795
	N3	1.34	2.43	1.89	2.90	21	0.95	0.650
	REM	2.11	3.21	0.77	1.71	21	− 1.69	0.526
Nap sleep	Wake	13.08	12.23	25.04	17.02	21	3.73	0.005
	SOL	12.87	12.10	23.82	17.22	21	3.17	0.009
	N1	3.03	1.59	2.37	1.91	21	− 1.41	0.261
	N2	20.23	9.10	11.41	10.42	21	− 3.61	0.005
	N3	3.35	6.32	2.31	4.30	21	− 0.93	0.438
	REM	4.92	8.27	3.96	6.32	21	− 0.63	0.534

Means and standard deviations (SD; n = 22) in min in the placebo and caffeine conditions of the nocturnal sleep episode (top), the last 10 min of nocturnal sleep (middle), and the nap sleep opportunity (bottom) are reported. SOL = sleep onset latency; N1, N2, N3, REM = NREM and REM sleep states. df = degrees of freedom.

*P*-values refer to Benjamini–Hochberg corrected post-hoc comparisons.

#### Pre-awakening sleep

Statistical analyses of sleep variables during 10 min before scheduled awakening revealed no significant main effect of ‘condition’ (F = 0.03; *p* > 0.05 η^2^ = 0.0001) (Fig. 6B; Table 1).

#### Nap sleep opportunity

The statistical analyses of the sleep variables in the nap opportunity 1 h after scheduled awakening revealed a significant ‘condition’ effect (F = 2.09; *p* < 0.01; η^2^ = 0.87), such that sleep onset latency and wakefulness after sleep onset were prolonged. In addition, the time spent in stage N2 was reduced in the caffeine condition when compared to the placebo condition (*p*_all_ < 0.01; Fig. 6C; Table 1).

## Discussion

Here we tested the in vivo drug release profile and efficacy to ameliorate morning sleep inertia of a delayed pulsatile-release caffeine formulation administered at bedtime. We found that this innovative approach potently facilitated the sleep-to-wake transition on neurobehavioral, subjective and physiological markers of sleep inertia in healthy young men following sleep restriction. Most importantly, in contrast to reactive caffeine intake, the novel formula improved vigilance within the first 15 min and mood at 15 min after waking in comparison to placebo.

The in vivo validation study corroborated the intended release profile. As expected based on the in vitro development of the engineered caffeine micropellets, the caffeine curve followed a delayed-release profile, and an efficacious plasma concentration above ~ 5 μM was attained only after 7 h. Unexpectedly, the release profile of the identical formulation in the pharmacodynamic study was distinctly different from the validation study. Premature caffeine-release started already 3.5 h after drug administration and efficacious plasma levels were attained already 5 h after drug intake. We suggest that a premature burst of the formula was probably triggered by the meal served to the subjects 3.5 h after caffeine administration (this meal was not served in the validation study). This food intake may have promoted gastric movements that caused faster gastric emptying and provoked the break of the release-modifying polymeric coat due to the physical impact of the peristalsis. Further studies are needed to confirm this hypothesis. In addition, future research may also clarify whether the pharmacokinetics of the engineered caffeine formula systematically differs between sleep and wakefulness.

Despite the premature release of caffeine, the formula ameliorated sleep inertia on the subsequent morning following only 4 h of sleep. The quality of awakening was subjectively improved on behavioral, cognitive, emotional, and physical levels, as indicated on all subscales of the sleep inertia questionnaire. Even though the study participants were sleep restricted, many of them reported less difficulty to rise when compared to a habitual morning, particularly in the caffeine condition. The formulation also exhibited mood enhancing properties, as indicated by increased positive and reduced negative ratings on the PANAS. This finding may suggest increased activity of mood-relevant brain structures in the caffeine condition when compared to the placebo condition. Caffeine blocks adenosine A_2A_ receptors in the nucleus accumbens^50,51^, a core region of the mesolimbic dopamine network that is essential for the generation of positive mood and reward^52^. We speculate that caffeine-induced dopamine release in the nucleus accumbens^53^ could contribute to the post-awakening mood enhancement when compared to placebo. Other possible mechanisms include increased preparatory attention for rewarding stimuli^54^ or direct interaction with sleep–wake regulatory pathways such as the circadian clock^55^. The mechanistic underpinnings of the benefit of the pulsatile caffeine-release formulation on subjective state will have to be clarified in future studies.

The arousal effect of caffeine relies on the competitive antagonism of central nervous system adenosine receptors that contribute to the regulation of sleep intensity and sleep need^14,50^. Sleep inertia, in particular upon sleep restriction, was previously proposed to reflect ‘adenosine left overs’ that were insufficiently removed during sleep^1,56^. Consistent with this view, the primary beneficial effects of the tested caffeine release formula were not restricted to subjective state but included improved vigilance as manifested by faster PVT reaction times when compared to placebo. This finding is important because after nocturnal sleep, no easy applicable proactive or efficacious reactive countermeasure to impaired neurobehavioral performance due to sleep inertia is currently available^6^. Previous work suggested reduced lapsing on the PVT immediately after waking from a 30-min coffee-nap ending at 04:00^24^ and from repeated 2-h sleep opportunities during prolonged sleep deprivation^16^. In the present study, we observed no changes in the number of PVT lapses. The differences in the experimental protocols, as well as the caffeine dosages and application forms may underlie the discrepancy. Furthermore, we found no improvement in cognitively more demanding tasks such as sustained selective attention and visual scanning speed (assessed with the d2-task) and working memory and executive functioning (n-back task). These tasks were administered > 45 min after waking. Rested baseline measurements would be necessary to determine whether performance on these tasks at the time of their administration was impaired by sleep inertia and could be improved with the intervention tested.

After caffeine, the cortisol level on the CAR was increased 1 h after wake-up when compared to placebo. This finding supports the hypothesis that caffeine stimulates cortisol secretion and augments the CAR upon waking. The wake-up-related cortisol secretion was previously suggested to reflect a hormonal wake-promoting signal^20^. Nevertheless, neither the peak cortisol concentration nor the area-under-the-curve were affected by caffeine, suggesting that a direct association between reduced sleep inertia within the first 15 min of waking and HPA-axis activity is rather unlikely. Recent studies in rats revealed that overexpression of adenosine A_2A_ receptors may contribute to glucocorticoid receptor dysfunctions in aged animals and that caffeine may re-sensitize glucocorticoid receptors in the hypothalamus and restore HPA-axis function^57^. Although the observed effect on the CAR in our young healthy sample was subtle, it may be speculated that a delayed-release caffeine formulation may promote cortisol release and glucocorticoid receptor functioning in susceptible individuals^58^.

Due to the sustained-release profile of the engineered formula, the blood caffeine concentration remained within an efficacious dose range until 17.5 h after administration. Consistent with this pharmacokinetic profile, the increased wake time after sleep onset, the prolonged sleep latency and the reduced N2 sleep duration during the 1-h nap after awakening support the notion that sustained low-dose caffeine administration improved post-awakening vigilance^16^. The premature high concentration of caffeine during nocturnal sleep most likely also underlies the ~ 17-min reduction in deep N3 sleep observed during the main sleep episode. Such a reduction in deep sleep would hamper the applicability of this novel pulsatile-release caffeine formula. Ongoing research employing quantitative sleep EEG analyses as a function of caffeine levels during sleep, as well as follow-up sleep studies will determine whether sleep is also disturbed without intra-night food intake. With respect to the reduced sleep depth, it seems unlikely that the mitigated sleep inertia after waking depended on the reduced duration of N3 sleep because this sleep state did not differ between the conditions during the final 10 min before scheduled awakening.

Taken together, this proof-of-concept investigation demonstrates that a timed pulsatile-release caffeine system ingested at bedtime can potently attenuate neurobehavioral, subjective, emotional and physiological manifestations of morning sleep inertia in sleep-restricted healthy young men. These findings cannot be generalized because only a single dose, only healthy men, and individuals irrespective of their caffeine sensitivity were studied. Nevertheless, if future research supports these conclusions and further improves the drug-release profile of the engineered formula, time-controlled caffeine administration may be developed as an add-on therapy to mitigate impaired morning state and vigilance in people suffering from excessive sleep inertia, which is highly prevalent in on-call and shift work settings, as well as in patients with neurological, neuropsychiatric, and circadian-rhythm sleep–wake disorders.
